# Stability of the Transthyretin Molecule as a Key Factor in the Interaction with A-Beta Peptide - Relevance in Alzheimer's Disease

**DOI:** 10.1371/journal.pone.0045368

**Published:** 2012-09-17

**Authors:** Carlos A. Ribeiro, Maria João Saraiva, Isabel Cardoso

**Affiliations:** 1 Molecular Neurobiology Unit, IBMC- Instituto de Biologia Molecular e Celular, Porto, Portugal; 2 ICBAS- Instituto de Ciências Biomédicas Abel Salazar, Porto Portugal; 3 Escola Superior de Tecnologia da Saúde do Porto, Instituto Politécnico do Porto, Vila Nova de Gaia, Portugal; Illinois Institute of Technology, United States of America

## Abstract

Transthyretin (TTR) protects against A-Beta toxicity by binding the peptide thus inhibiting its aggregation. Previous work showed different TTR mutations interact differently with A-Beta, with increasing affinities correlating with decreasing amyloidogenecity of the TTR mutant; this did not impact on the levels of inhibition of A-Beta aggregation, as assessed by transmission electron microscopy. Our work aimed at probing differences in binding to A-Beta by WT, T119M and L55P TTR using quantitative assays, and at identifying factors affecting this interaction. We addressed the impact of such factors in TTR ability to degrade A-Beta. Using a dot blot approach with the anti-oligomeric antibody A11, we showed that A-Beta formed oligomers transiently, indicating aggregation and fibril formation, whereas in the presence of WT and T119M TTR the oligomers persisted longer, indicative that these variants avoided further aggregation into fibrils. In contrast, L55PTTR was not able to inhibit oligomerization or to prevent evolution to aggregates and fibrils. Furthermore, apoptosis assessment showed WT and T119M TTR were able to protect against A-Beta toxicity. Because the amyloidogenic potential of TTR is inversely correlated with its stability, the use of drugs able to stabilize TTR tetrameric fold could result in increased TTR/A-Beta binding. Here we showed that iododiflunisal, 3-dinitrophenol, resveratrol, [2-(3,5-dichlorophenyl)amino] (DCPA) and [4-(3,5-difluorophenyl)] (DFPB) were able to increase TTR binding to A-Beta; however only DCPA and DFPB improved TTR proteolytic activity. Thyroxine, a TTR ligand, did not influence TTR/A-Beta interaction and A-Beta degradation by TTR, whereas RBP, another TTR ligand, not only obstructed the interaction but also inhibited TTR proteolytic activity. Our results showed differences between WT and T119M TTR, and L55PTTR mutant regarding their interaction with A-Beta and prompt the stability of TTR as a key factor in this interaction, which may be relevant in AD pathogenesis and for the design of therapeutic TTR-based therapies.

## Introduction

Alzheimer's disease (AD) is nowadays responsible by 50 to 80 percent of dementia cases [1] and is mainly characterized by two types of lesions: neurofibrillary tangles (NFTs) and neuritic plaques. NFTs [2] are bundles of abnormal filaments composed of highly phosphorylated tau protein [3]. Neuritic plaques are extracellular amyloid deposits found in the brain and are mainly constituted by beta-amyloid (A-Beta) peptide. Accumulation of A-Beta is due to disregulated proteolytic processing of its precursor, the Amyloid Precursor Protein (APP).

Schwarzman *et al.* described that normal cerebrospinal fluid (CSF) inhibits amyloid formation [4] and concluded that transthyretin (TTR) was the major A-Beta binding protein in CSF, leading to a decrease in the aggregation state of the peptide [4]. More recently it has been shown that deletion of the TTR gene in a mouse APP transgenic model results in increased of A-Beta deposition [5]. Our group characterized the TTR/A-Beta interaction showing that TTR is capable of interfering with A-Beta fibrilization by both inhibiting and disrupting fibril formation [6]. We also demonstrated that TTR, either recombinant or isolated from human sera, was able to proteolytically process A-Beta. Furthermore, A-Beta new peptides (1–14) and (15–42), generated upon cleavage by TTR, showed lower amyloidogenic potential than the full length counterpart [7].

TTR is a homotetramer and it is thought that the first step in the cascade that results in amyloid formation, is the dissociation of the tetramer into monomers [8]. TTR is responsible for the transport of thyroxine (T_4_) and retinol, through binding to the retinol binding protein (RBP). The four monomers within a TTR tetramer, form an open channel where T_4_ binds [9], [10] while retinol interacts with only one of the dimers, at the surface [11]. TTR, when mutated, is associated to another amyloidotic disorder, Familial Amyloid Polyneuropathy (FAP), characterized by the extracellular deposition of TTR in several organs with a special emphasis in the peripheral nerve.

Particular mutations in the protein lead to instability of the native fold, increasing its propensity to precipitate and aggregate. For instance, it was suggested that mutated L55P TTR significantly alters tetramer stability and increases amyloidogenicity [12]. The authors described that L55P TTR tetramer was more unstable than the WT TTR and that the ability of L55P TTR to develop to an amyloidogenic intermediate occurred at higher pHs than the wild-type protein; this could explain why some TTR variants form amyloid fibrils, while the WT TTR remains stable and nonamyloidogenic [12]. Furthermore, Longo Alves *et al.* compared the stability and clearance of V30M TTR and T119M TTR and described that the more stable properties of T199M variant could be involved in the protective clinical effect of the T119M mutation in FAP. Baures *et al.* and Oza *et al.*, demonstrated several small compounds sharing molecular structural similarities with TTR natural ligand − T_4_ − that can bind in the T_4_ binding sites, proposing them as inhibitors of TTR fibril formation *in vitro* [13], [14]. In addition and related to the stabilization of TTR, Costa *et al.* described that TTR mutations, such T119M, Y78F, V30M and L55P bound differently to A-Beta. Additionally, an inverse relation between the amyloidogenic potential of TTR and the affinity to A-Beta peptide was suggestive of a direct relationship with TTR stability [6].

In the present work we further explore TTR/A-Beta interaction, in particular the influence of TTR stability and ligands, on binding properties.

## Materials and Methods

### TTR production and purification

Recombinant TTRs were produced in a bacterial expression system using *Escherichia coli* BL21 [15] and purified as previously described [16]. Briefly, after growing the bacteria, the protein was isolated and purified by preparative gel electrophoresis after ion exchange chromatography. Protein concentration was determined using the Bio-Rad assay kit (Bio-Rad), using bovine serum albumin (BSA) as standard.

### Production of A-Beta species

The peptide was purchased from Genscript, dissolved in Hexafluoroisopropanol (HFIP) and kept at room temperature overnight. The HFIP was removed under a stream of nitrogen and the residue was then dissolved in Dimethyl sulfoxide (DMSO) at 2 mM. For experiments, A-Beta was diluted in the buffers mentioned below at the indicated concentrations.

### A11 antibody assay

Soluble A-Beta alone (100 µM) or mixed with WT TTR, T119M or L55P TTR (10 µM), or TTR alone, were incubated at 37°C and aliquots of 0.45 µg A-Beta were removed over time and immobilized onto a nitrocellulose membrane. Detection of oligomers was performed using the A11 antibody (1∶500, Biosource), followed by an anti-rabbit HRP (horseradish peroxidase) conjugate (1∶500) as the secondary antibody. Detection was performed with ECL® (enhanced chemiluminescence; GE Healthcare).

### Cell culture and caspase-3 assay

SH-SY5Y cells (human neuroblastoma cell line; (European Collection of Cell Cultures)) [17], were propagated in 25-cm flasks and maintained at 37°C in a humidified atmosphere of 95% and 5% Carbon dioxide (CO_2_) and grown in MEM/F12 (1∶1) (Lonza) supplemented with 15% fetal bovine serum (FBS) (Gibco BRL). Activation of caspase-3 was measured using the CaspACE fluorimetric 96-well plate assay system (Sigma), following the manufacturer's instructions. Briefly, 80% confluent cells were cultured in 6-well plates and were grown for 24 hours. Then, the medium was renewed 3–5 hours later, and 10 µM A-Beta peptide, previously incubated with and 2 µM TTR, WT, T119M or L55P, for 48 hours at 4°C in F12 media (Lonza), was added to the cells. Subsequently, each well was trypsinized and the cell pellet was lysed in 100 µl of hypotonic lysis buffer (Sigma). 40 µl of each cell lysate was used in duplicates for determination of caspase-3 activation. The remaining cell lysate was used to measure total cellular protein concentration with the Bio-Rad protein assay kit (Bio-Rad), using BSA as standard. Experiments were repeated at least twice; values shown are the mean of duplicates.

### Chemical Compounds

Tri-iodophenol (TIP), Flufenamic Acid (Fluf), Diflunisal (Dif), Resveratrol (Resv), 2-((3,5-Dichlorophenyl)amino)benzoic acid (DCPA), Dinitrophenol (DNP), 4-(3,5-difluorophenyl) (DFPB), Genistein, Epigallocatechin gallate (EGCG) and thyroxine (T_4_) were from Sigma. The diflunisal derivative, iododiflunisal (I-Dif, Mr 376.1) was kindly provided by Dr. Gregorio Valencia, CSIC, Barcelona [18]. Retinol Binding Protein (RBP) was isolated from serum by affinity in a TTR column and saturated with 3.3 mg/ml all-*trans* retinol (Sigma) in ethanol as follows: (i) 25 µl of all-*trans*-retinol were incubated with 800 µl of RBP (1 mg/ml) at 37°C in the dark for 1 h; and (ii) excess retinol was separated from RBP by gel filtration in 10-ml Biogel P-6 DG columns (Bio-Rad)[19].

### Competition binding assays

Recombinant TTRs were iodinated with Na^125^I (NEN) using the Iodogen (Sigma) method, following the supplier's instructions. The reaction was desalted by Sephadex G50 gel filtration; 96 well plates (Maxisorp, Nunc) were coated with A-Beta (5 µg/well) in coating buffer (Na_2_CO_3_/NaHCO_3_ pH 9.0) and incubated overnight at 4°C. Unoccupied sites were blocked with 5% non-fat dried milk in PBS for 2 hours at 37°C. For competition studies, different concentrations of cold TTR (0× to 100× molar excess), alone or pre-incubated for 12 hours with a 5× molar excess of RBP or different compounds included: TIP, Fluf, Dif, I-Dif, Resv, DCPA, DNP, DFPB, Genistein and EGCG were used and mixed with a constant amount of ^125^I-WT TTR (500000 cpm) and incubated for two hours at 37°C. Binding was determined after five washes in ice cold phosphate-buffered saline with 0.05% tween20 (PBS-T). Then, 0.1 ml of elution buffer (NaCl 0.1 M containing Nonidet P40 (NP40) 1%) was added for 5 minutes at 37°C and the contents of the wells aspirated and counted in a rackgamma counter. Experiments were repeated at least twice in quadruplicates.

### A-Beta proteolysis assay

A fluorogenic peptide encompassing 6 residues of the A-Beta peptide sequence containing one of the TTR cleavage sites previously described [7] was used (Abz-VHHQKL-EDDnp, Genscript); this substrate is an internally quenched fluorescent peptide in which Abz (*ortho*-aminobenzoic acid) is the fluorescent donor and EDDnp [*N*-(ethylenediamine)-2,4-dinitrophenyl amide] is the fluorescent quencher. Hydrolysis of the fluorogenic substrate was monitored by measuring fluorescence at λ_em_ = 420 nm and λ_ex_ = 320 nm in a *f*max plate reader (Molecular Devices). The kinetics of the reaction was followed for 1 h at 37°C. To determine the effect of different TTR ligands on its ability to cleave A-Beta peptide, 5 µM of TTR was pre-incubated (for 1 hour) with the compounds described above (till 10 fold molar excess) at 37°C with 50 mM Tris pH 7.5 and then 5 µM of substrate was added in a final volume of 100 µL. Experiments were repeated at least three times.

### Assessment of tetrameric TTR stability by IEF (isoelectric focusing) in semi-denaturing conditions

The conditions used for the IEF of plasma TTR have been described previously by Altland et al. [20] and Almeida et al. [18]. Briefly, 12 µg of recombinant L55P TTR were incubated at 4°C overnight, with 10 mM solution of the tested compounds. The preparations were then subjected to native PAGE to isolate TTR. The gel band containing TTR was excised and subjected to IEF in a gel with 4 M urea and 5% ampholytes (pH 4–6.5) at 1200 V for 6 h. Proteins in the gel were stained with Coomassie Blue. These semi-denaturing conditions allow the visualization of bands corresponding to the TTR monomer and tetramer and also to an oxidized form of the monomer. Gels were scanned and bands were quantified using the Quantity One program. Experiments were repeated at least two times and data shown are representative of the results obtained.

### Statistical analysis

All data were expressed as mean values ± standard error of the mean (SEM). One way ANOVA with Bonferroni's post-test was performed using GraphPad Prism, version 5.04 for Windows, GraphPad Software, San Diego California USA, www.graphpad.com. Values of p<0.05 were considered to be significant.

## Results

### 1. Differences in binding to A-Beta peptide by WT, T119M and L55P TTRs using quantitative assays

#### a. Effect on A-Beta peptide oligomerization

Previous results indicated that TTR mutants bind differently to A-Beta peptide, with the following trend: T119M > WT > V30M ≥ Y78F > L55P TTR, suggesting that TTR amyloidogenecity influences its binding to the peptide; at the level of A-Beta aggregation, we did not detect any differences between TTR mutants, as both non-amyloidogenic and amyloidogenic variants were able to prevent A-Beta fibrilization with approximately the same extent, as evaluated by transmission electron microscopy (TEM) [6]. In the present work, we further compared if different TTR variants differently affect A-Beta fibrillogenesis, making use of a dot blot assay with the A11 antibody, described as specific for oligomeric species. This antibody has the ability to recognize an amyloidogenic conformation present in intermediate species from different amyloidogenic precursors; however, it does not recognize their soluble, aggregated or fibrillar counterparts [21].

**Figure 1:**
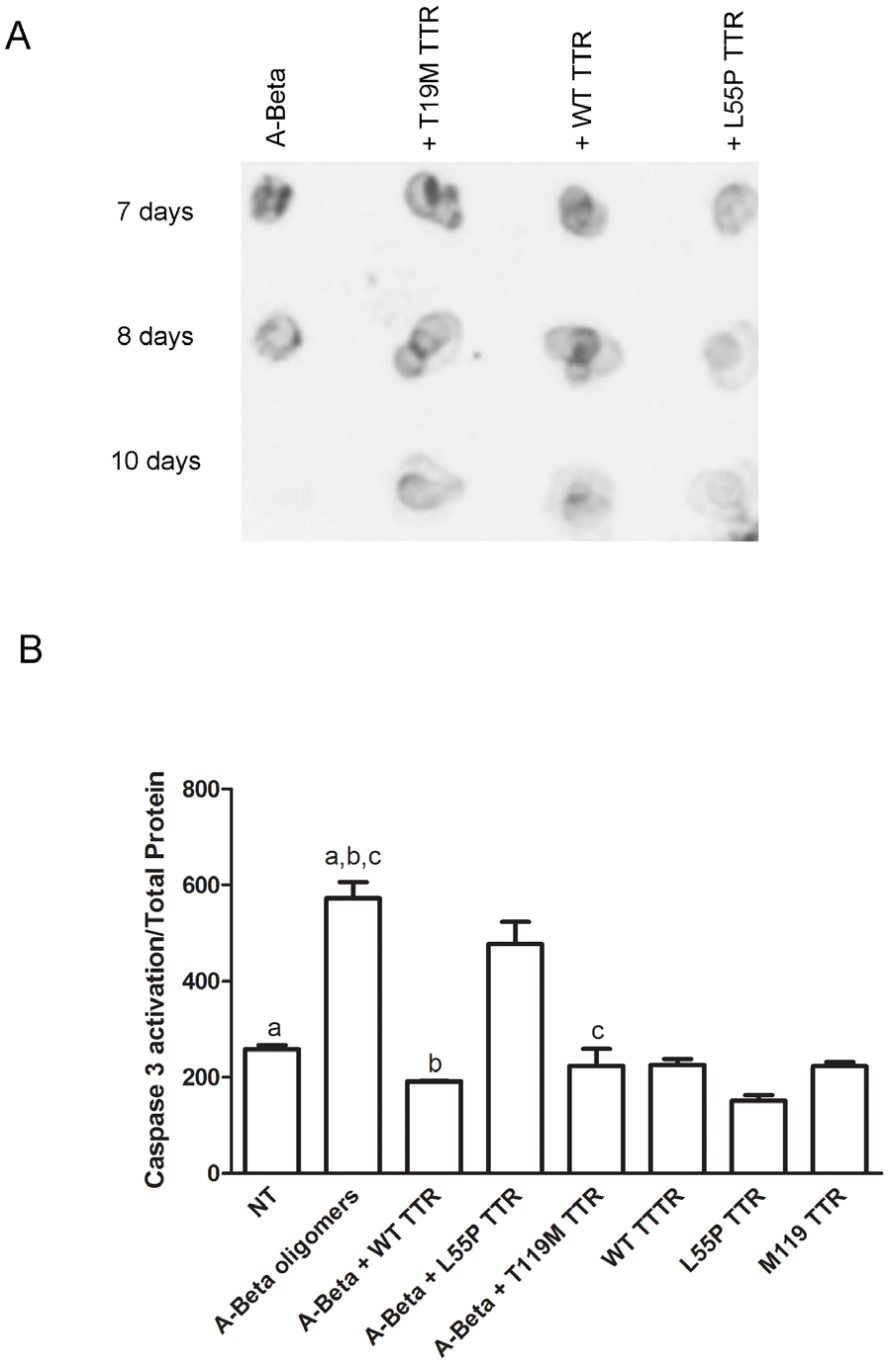
Differences in binding to A-Beta peptide by WT, T119M and L55P TTRs variant using quantitative assays. A- Immunodot blot of A-Beta alone or mixed with WT TTR, T119M or L55P TTR, incubated at 37°C for different periods of time, and analyzed with the anti-oligomeric antibody A11. WT and T119M TTRs interfered with A-Beta oligomerization resulting prolonged presence of oligomers, compared to A-Beta alone which evolved to aggregates and fibrils. In opposition, L55P TTR had impaired ability to avoid fibrillization of the peptide. **B-** Caspase 3 activation in SH-SY5Y cells. A-Beta without or with TTR (WT TTR, T119M or L55P TTR) incubated at 4°C for 48 h, was then added to SH-SY5Y cultured cells and further incubated for another 48 h at 37°C. A-Beta was used at a final concentration of 10 µM and TTR at 2 µM. Significant caspase 3 activation was observed in the presence of A-Beta peptide alone and when pre-incubated with L55P TTR. WT and T119M were able to protect against A-Beta toxicity and lower levels of caspase 3, similar to untreated cells, were observed. ^a,b,c^p<0,001

Soluble A-Beta alone or mixed with WT, TT119M or L55P TTR mutant were incubated at 37°C and aliquots of 0.45 µg were removed over time and immobilized onto a nitrocellulose membrane. For the incubation of A-Beta alone, detection with the A11 antibody showed the presence of A-Beta oligomers starting at day 0 (not shown) which continued until day 8 (figure 1A). At day 10 the A11 signal disappeared completely, suggesting that A-Beta oligomers evolved to other species such as aggregates and fibrils which are not recognized by this antibody. Co-incubation of the peptide with WT TTR produced a different profile as the presence of oligomers was prolonged until at least day 10 (last time point analysed) (figure 1A), indicative that the WT protein avoided further aggregation of the peptide into fibrils, by arresting the oligomer stage. TTR T119M, a protective mutation in FAP patients, was also able to avoid further aggregation of A-Beta peptide, producing an A-Beta profile similar to the one treated with WT TTR.

On the contrary, L55P TTR was not able to inhibit oligomerization nor to prevent evolution to aggregates and fibrils (figure 1A), resulting in a fainter A11 signal in the last days analyzed, indicating that A-Beta aggregated and evolved to aggregates and fibrils. The same amount of WT, T119M and L55P TTR were also incubated alone and analysed over time, as above; detection with the A11 antibody revealed no reaction confirming that the signal observed previously was from A-Beta (not shown).

#### b. Effect on A-Beta toxicity

We next analyzed the effect of TTR on A-Beta toxicity, using the same TTR variants, WT T119M and L55P TTR. Caspase 3 activation was measured in SH-SY5Y cells treated with A-Beta previously oligomerized for 48 hours, alone or in the presence of the selected TTR variant; controls of TTRs alone were also included. Figure 1B shows that WT TTR was able to prevent the toxicity associated to A-Beta oligomers, as previously shown [6]; Moreover, the non-amyloidogenic variant T119M also significantly inhibited caspase 3 activation, therefore preventing A-Beta toxicity, whereas A-Beta pre-incubated with L55P TTR produced similar levels of active caspase 3 as found in cells treated with A-Beta alone.

Altogether, the oligomerization and toxicity studies indicated differences between the non-amyloidogenic TTR variants WT and T119M, and the highly amyloidogenic mutant L55P TTR regarding their ability to interfere with A-Beta peptide amyloidogenic properties.

### 2. Importance of TTR stability in its interaction with A-Beta peptide

#### a. Effect of TTR stabilizers in A-Beta binding

We tested whether small compounds known to stabilize the TTR tetrameric fold should restore the capacity of TTR to bind A-Beta, in particular the L55P TTR variant with low binding capacity. To test this hypothesis, we used competition binding assays as this technique allows not only the assessment of the interaction between molecules but also to infer on the strength of the interaction. We started by using Dif, a non-steroid anti-inflammatory (NSAIDs), and its derivative, I-Dif. The latter has been shown to highly stabilize the TTR tetramer structure, *in vitro* and *ex vivo*, whereas Dif could only produce a mild effect, under the same conditions. [18].

**Figure 2:**
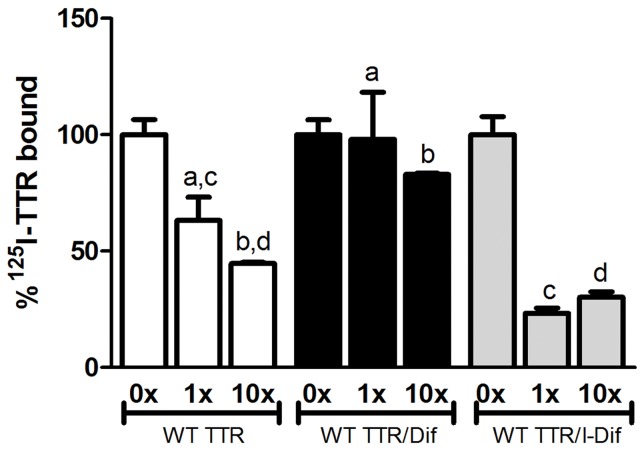
Effect of small compounds on WT TTR binding to A-Beta peptide. A-beta peptide immobilized onto a microtiter plate was incubated with a constant amount of ^125^I-WT TTR and increasing concentrations of unlabeled WT TTR, and binding was evaluated. Alternatively and to investigate the ability certain compounds to improve TTR/A-Beta interaction, unlabeled TTR was pre-incubated with I-Dif or Dif, and further added to the immobilized A-Beta peptide. I-Dif, but not Dif, was able to improve TTR/A-Beta interaction. ^a^p<0.05; ^b^p<0.05; ^c^p<0.05, ^d^p<0.05.

Using competition binding assays after pre-incubation of WT TTR with the referred drugs, we observed that the presence of I-Dif improved TTR binding to A-Beta peptide, whereas Dif, on the contrary, worsened the binding (figure 2). Other small compounds known to stabilize the TTR tetrameric fold such as Resv, DNP, DCPA, DFPB, EGCG, flufenamic acid, genistein, benzo and TIP [22] were also assayed but we did not observe improvement in the WT TTR/A-Beta binding (data not shown).

**Figure 3:**
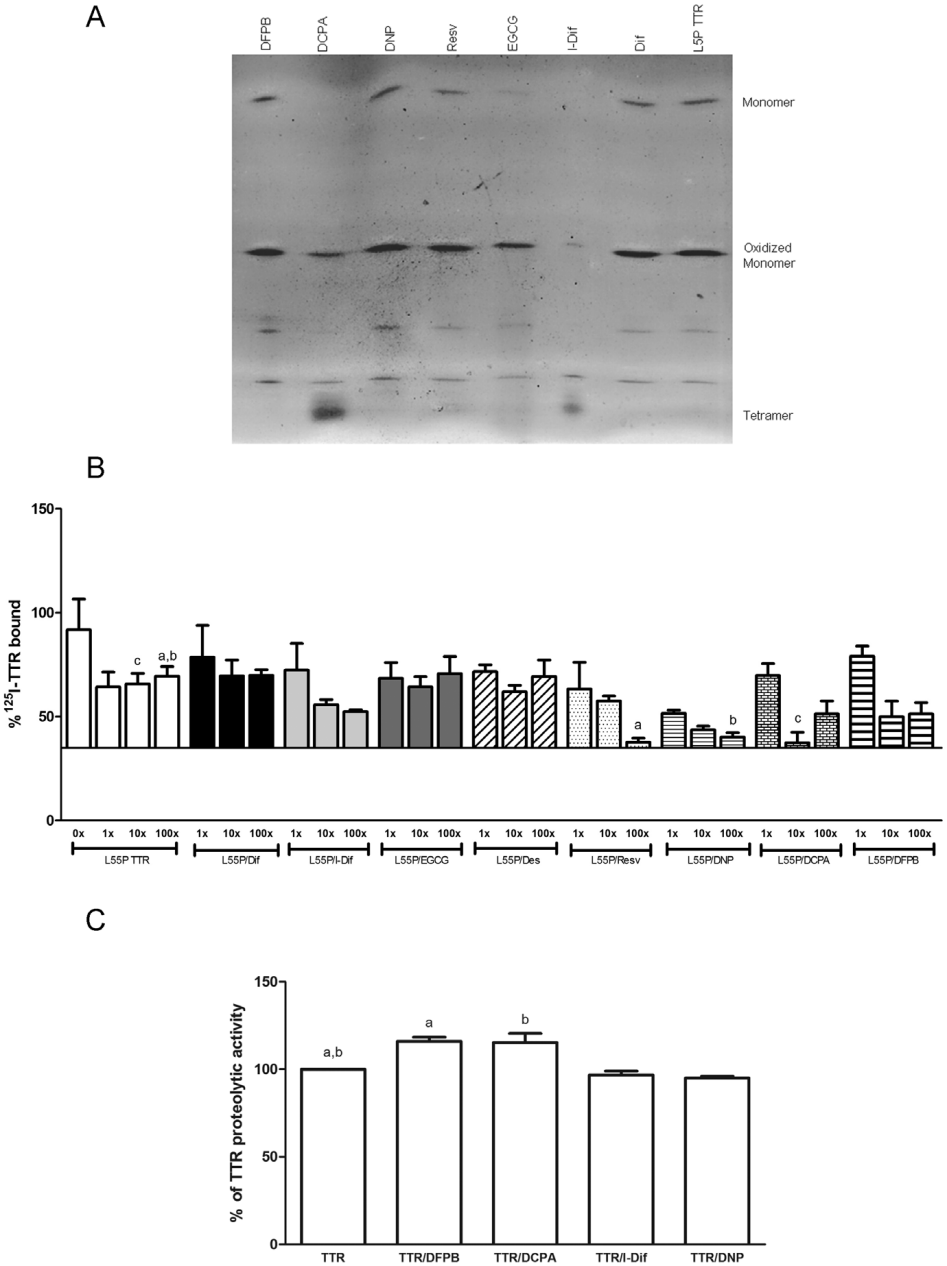
Effect of small compounds on L55P TTR binding to A-Beta peptide and consequences in TTR proteolytic activity. A-IEF analysis of L55P alone or in the presence of different compounds known as TTR stabilizers. Similarly to previous results obtained with WT and V30M TTR (22, 23), DCPA and I-Dif were able to stabilize the L55P fold, resulting in increased amounts of tetramer and decreased quantity of monomer; compounds such as DFPB, DNP, Resv, EGCG and Dif did not produce any effect and levels of tetramer and monomer were similar to the L55P protein alone. **B**- A-beta peptide immobilized onto a microtiter plate was incubated with a constant amount of ^125^I-L55P TTR and increasing concentrations of unlabeled L55P TTR, and binding was evaluated. Alternatively and to investigate the ability certain compounds to improve TTR/A-Beta interaction, unlabeled TTR was pre-incubated with I-Dif or Dif, EGCG, resv, DNP, DCPA and DFPB, and further added to the immobilized A-Beta peptide. Using this variant, I-Dif, Resv, DNP, DCPA and DFPB were selected as improving TTR/A-Beta interaction, whereas Dif, EGCG and DES had no effect. ^a^p<0.05; ^b^p<0.05; ^c^p<0.05. C- Effects of selected compounds on A-Beta proteolysis by TTR, showing that DCPA and DFPB improved the TTR proteolytic activity by approximately 15%, whereas other compounds such as I-Dif and DNP produced no effect. ^a,b^p<0.05

We reasoned that drugs with more modest effects, not as striking as I-Dif, may not produce visible impact on WT TTR/A-Beta binding, but could produce a profound effect if applied to the L55P TTR variant. We started by performing IEF assays to investigate if this variant was also stabilized by the same compounds as the WT and V30M counterparts did [22], [23], and found no differences in the degree of stabilization among the three proteins (i.e tetramer/monomer ratio) for the different tetramer stabilizing drugs (figure 3A). We then repeated the A-Beta binding assays with L55P and, as expected, this variant bound poorly to the peptide since no differences in binding were observed in the presence of the different cold TTR concentrations used (Figure 3A, white bars). Nevertheless, in our assays we detected a decrease in binding when comparing ^125^I-TTR L55P alone to ^125^I-TTR L55P mixed with the different cold TTR concentrations, which may reflect interference from the ^125I^ atom. On the contrary, WT TTR clearly bound A-Beta peptide as concluded by the increased level of competition between ^125^I-TTR and increasing cold TTR concentrations (figure 2).

After drug pre-incubation with TTR L55P we observed increased TTR/A-Beta interaction using DCPA, DFPB, Resv and DNP similarly to the positive control (I-Dif) (figure 3B). However, Dif (negative control) and EGCG persisted failing to produce any effect (figure 3B), as well as other compounds such as flufenamic acid, genistein, benzo and TIP (data not shown).

For some of the compounds, being particularly pronounced for DCPA, there is a marked increase in binding at higher concentrations (100x). Usually, this high concentration is used to assess non-specific binding.

#### b. Effect of TTR stabilizers in A-Beta proteolysis

It has been previously shown in our laboratory that TTR is able to proteolytically process the A-Beta peptide, contributing to reduction of its toxicity, as the generated peptides are less amyloidogenic than the full-length peptide [7]. This finding suggested A-Beta proteolysis by TTR as a protective mechanism in AD.

Because it is not known if binding to A-Beta peptide and its degradation by TTR is part of the same protective mechanism, it was important to assess the impact of the above selected drugs on TTR ability to degrade A-Beta, thus ascertaining if TTR stability is directly related to its proteolytic function.

Using a fluorescent peptide containing part of the A-Beta sequence and one of the TTR cleavage sites described for A-Beta [7], we observed that only DCPA and DFPB showed increase of 15% in TTR proteolytic ability to degrade A-Beta peptide (figure 3C) whereas the remaining, like I-Dif, DNP (figure 3C), Dif, Resv, EGCG, flufenamic acid, genistein, benzo and TIP, produced no effect (data not shown).

Our results suggested TTR stability as a key factor in the interaction with A-Beta. Proteolysis can also be modulated by small compounds known to affect TTR stability/aggregation; however, different drugs have different effects at the binding and at the proteolysis levels, suggesting that these are separate events or that the TTR regions involved, in the two activities, are different.

### 3. Effect of TTR natural ligands on TTR binding to A-Beta and impact in A-Beta proteolysis

**Figure 4:**
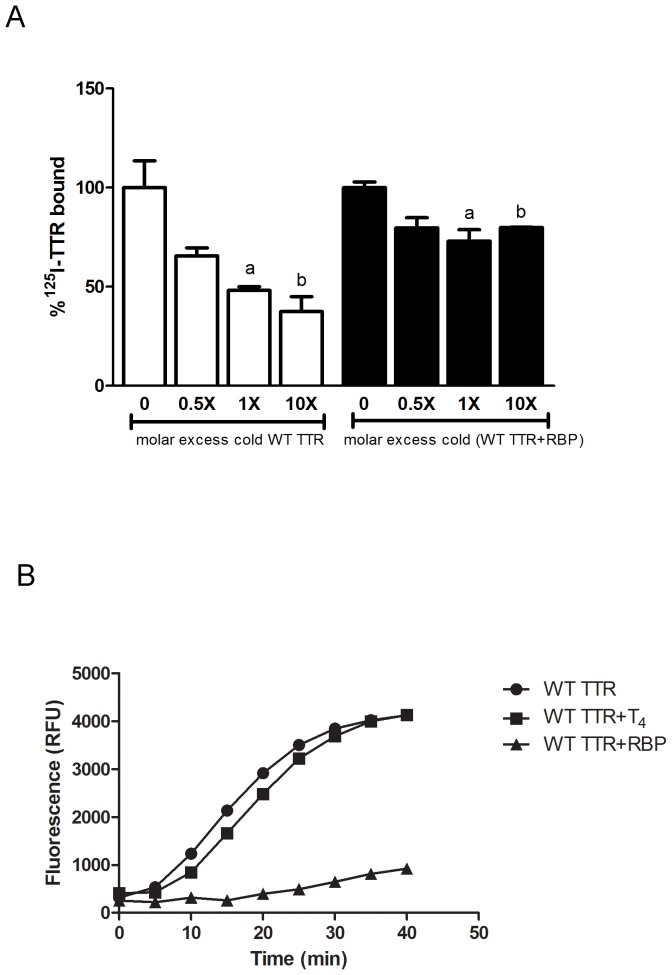
Effect of RBP on TTR binding to A-Beta peptide and consequences of T_4_ and RBP in TTR proteolytic activity. A - Competition binding assay between A-Beta and ^125^I-TTR in the presence of increasing concentrations of cold TTR or cold TTR saturated with RBP, showing that the presence of RBP abrogated binding. **B-** Effect of T_4_ and RBP in A-Beta degradation by TTR using a fluorescent peptide containing an A-Beta sequence. Similarly to binding, TTR proteolytic activity is abolished in the presence of RBP, while T_4_ does not interfere with this activity.

Previous work by competition binding assays showed that A-Beta peptide did not compete with T_4_ for binding to TTR [6]; here we show that, on the contrary, A-Beta displayed decreased binding to TTR saturated with RBP, as no significant competition was observed between ^125^I-WT TTR and cold (WT TTR+RBP) (figure 4A).

We next investigated the impact of the natural ligands (i.e. T_4_ and RBP) in the proteolytic capacity of TTR to degrade A-Beta peptide. We found that T_4_ did not affect the TTR ability to cleave A-Beta peptide; in contrast, when TTR is complexed with RBP, it drastically reduced its capacity to cleave A-Beta peptide, in as much as 82% (figure 4B).

## Discussion

Apart from its function as a carrier of T_4_ and retinol, TTR has taken more recently a very important role in amyloidogenic diseases and it is one of the 30 human proteins associated with amyloidosis, in particularly FAP. Although, clinically, TTR concentration in serum can be utilized as a marker of nutritional/inflammatory status [24] its reduction in CSF and plasma has been associated with AD development [25], [26]. In fact, TTR has been described as the major A-Beta binding protein in CSF avoiding its aggregation and toxicity [27], [28]. Furthermore, investigation using *in vivo* models has established TTR as a survival molecule since increased levels of TTR are observed in transgenic mice for APP [29]. Moreover, disruption of the TTR gene results in increased and earlier A-Beta deposition [5]. However, the mechanism by which TTR protects against A-Beta toxicity, the pathways and molecules involved are not identified. Recently, we proposed that TTR can proteolytically process A-Beta peptide, generating smaller and less amyloidogenic new peptides enabling cells to eliminate them [7].

Previous work, using qualitative assays, established that different TTR variants bind A-Beta peptide with different affinities; however no differences were found in their capacity to avoid A-Beta aggregation. In the present work, using quantitative assays, we showed differences between WT and T119M TTR and the less stable L55P TTR mutant regarding their interaction with A-Beta. We described that L55P TTR, contrarily to the WT and T119M counterparts, when incubated with A-Beta peptide was not able to prevent A-Beta aggregation and fibrillization; in addition, this variant was also not capable of avoiding A-Beta oligomers toxicity.

One of the therapeutic strategies for TTR-related amyloidosis aims at stabilizing the TTR tetrameric fold using small molecules which can either i) bind the T_4_ binding central channel or ii) bind outside the T_4_ binding channel. In group I, drugs such as iododiflunisal [18], TIP [30], DCPA and DFPB (Almeida, MR., personal communication, 2011), diclonisal [31], benzoxazole, DES [32], flufenamic acid [33], [34], DNP [35] and genistein [36] have been identified. Previous work by Cardoso et al [22] showed that TTR stabilization may be achieved by mechanisms other that binding to the T_4_ channel (group II) and compounds such as diclofenac [18], resveratrol [32], diflunisal [18], daidzein [37] were identified to avoid TTR aggregation in cellular studies [22] and in *in vivo* studies using an FAP mice model [38], [39].

We reasoned that stabilization of TTR by these two types of compounds could also improve binding to A-Beta. If true, poor TTR/A-Beta binding of L55P TTR would be rescued by TTR stabilizing drugs. For compounds belonging to group I, we used I-Dif and Dif as controls, positive and negative, respectively. Our hypothesis was confirmed as, in fact, DCPA - a strong T_4_ competitor - was also able to significantly improve TTR/A-Beta binding. We also investigated the effect of drugs belonging to group II (non-T_4_ competing drugs) on TTR/A-Beta binding and found, among the compounds tested that resv also increased TTR/A-Beta interaction, further confirming our hypothesis. DFPB was shown to compete moderately with T_4_ for TTR binding (Almeida M.R., personal communication, 2011), but was able to improve TTR/A-Beta interaction as much as DCPA and I-Dif. DFPB has been shown to greatly avoid TTR aggregation using a cellular model [22], however, by IEF, DFPB does not produce the expected increase in tetramer/monomer TTR ratio [22] and thus its TTR-stabilizing properties must be tetramer-independent. To further understand how DFPB stabilizes TTR, it is important to determine the structure of the complex.

Recently, it has been shown that EGCG is able to avoid TTR aggregation *in vitro and in vivo* [40], [41] and to disaggregate pre-formed TTR amyloid fibrils [23]. Although EGCG does not compete with T_4_, its binding at the surface of the protein results in TTR tetrameric stabilization as assessed by IEF [40]. In fact, Miyata et al. determined the crystal structure of the EGCG-V30M TTR complex and showed that there are three EGCG binding sites in TTR that are not related with T_4_ binding sites. Although, it is not clear which residues are crucial for EGCG/TTR binding, they described that EGCG binds in the interface of both dimers, resulting in stabilization of the tetramer [42]. In the present studies, EGCG as well as other tested compounds had no effect or even worsened TTR/A-Beta binding, which can be explained if these drugs bind in or near the A-Beta binding in TTR, thus masking their ability to improve TTR stability and consequent increase in A-Beta binding.

To further ascertain the influence of stability in the role of TTR in AD, namely on proteolysis, we quantified TTR proteolytic activity after incubation with a fluorogenic peptide encompassing 6 residues of the A-Beta peptide sequence containing one of the TTR cleavage sites as previously described [7]. In our assay, only DCPA and DFPB facilitated A-Beta proteolysis by TTR, raising the hypothesis that binding and proteolysis are two distinct mechanisms through which TTR protects against AD. At this point there is no information neither on the TTR region that binds A-Beta nor on the residues important for its proteolytic activity, which was firstly described for ApoAI [19] and more recently for NPY [43]. Possibly, the two events are directly related, and depend on each other (for instance binding preceding proteolysis), but with different regions of the TTR molecule responsible for the two activities.

TTR natural ligands showed different effects in TTR binding and proteolytic activity towards A-Beta peptide. While T_4_ did not affect TTR/A-Beta interaction [6] and TTR proteolytic activity, RBP not only prevented TTR binding to A-Beta but also abolished TTR ability to cleave the peptide. Liz et al. using a fluorogenic substrate corresponding to an apoA-I fragment encompassing amino acid residues 223–228, established that while TTR proteolytic activity was only slightly affected when complexed with T_4_, TTR complexed to RBP lost its proteolytic activity [19]. Because not all TTR is bound to RBP (the ratio RBP: TTR in plasma is approximately 0.3 in healthy individuals indicating that most of the circulating TTR remains free of ligand [44], [45]), it is expected that TTR proteolytic activity is not affected by RBP *in vivo*.

In summary, this work showed that mutations in TTR affect its ability to bind A-Beta peptide, interfere with A-Beta aggregation, and its noxious effects, at the cellular level. TTR protection in AD is probably dependent on its stability, and although genetic tests performed so far did not reveal mutations in the TTR gene of AD patients [46], destabilization of the protein may result from other events, such as metal ions concentration and interaction with other proteins. Small compounds known as TTR tetrameric stabilizers are able to improve TTR/A-Beta interaction; proteolysis also participates in TTR protection against A-Beta toxicity and may also be modulated by small TTR stabilizers.

These results may be of relevance for the design of therapeutic drugs that might improve TTR/A-Beta binding and TTR proteolytic capacity to decrease or inhibit A-Beta deposition in AD.
